# Context‐dependent integrated stress resistance promotes a global invasive pest

**DOI:** 10.1111/1744-7917.13035

**Published:** 2022-04-16

**Authors:** Vimbai L. Tarusikirwa, Ross N. Cuthbert, Reyard Mutamiswa, Casper Nyamukondiwa

**Affiliations:** ^1^ Department of Biological Sciences and Biotechnology Botswana International University of Science and Technology Palapye Botswana; ^2^ GEOMAR Helmholtz‐Zentrum für Ozeanforschung Kiel Kiel Germany; ^3^ School of Biological Sciences Queen's University Belfast Northern Ireland United Kingdom; ^4^ Department of Zoology and Entomology University of the Free State Bloemfontein South Africa; ^5^ Tugwi‐Mukosi Multidisciplinary Research Institute Midlands State University Gweru Zimbabwe; ^6^ Department of Zoology and Entomology Rhodes University Makhanda South Africa

**Keywords:** acclimation, cross‐talk, cross‐tolerance, invasive species, thermal tolerance, tomato leaf miner

## Abstract

In nature, insects concurrently face multiple environmental stressors, a scenario likely increasing with climate change. Integrated stress resistance (ISR) thus often improves fitness and could drive invasiveness, but how physiological mechanisms influence invasion has lacked examination. Here, we investigated cross‐tolerance to abiotic stress factors which may influence range limits in the South American tomato pinworm—a global invader that is an ecologically and socially damaging crop pest. Specifically, we tested the effects of prior rapid cold‐ and heat‐hardening (RCH and RHH), fasting, and desiccation on cold and heat tolerance traits, as well as starvation and desiccation survivability between *T. absoluta* life stages. Acclimation effects on critical thermal minima (CT_min_) and maxima (CT_max_) were inconsistent, showing significantly deleterious effects of RCH on adult CT_max_ and CT_min_ and, conversely, beneficial acclimation effects of RCH on larval CT_min_. While no beneficial effects of desiccation acclimation were recorded for desiccation tolerance, fasted individuals had significantly higher survival in adults, whereas fasting negatively affected larval tolerances. Furthermore, fasted and desiccation acclimated adults had significantly higher starvation tolerance, showing strong evidence for cross‐tolerance. Our results show context‐dependent ISR traits that may promote *T. absoluta* fitness and competitiveness. Given the frequent overlapping occurrence of these divergent stressors, ISR reported here may thus partly elucidate the observed rapid global spread of *T. absoluta* into more stressful environments than expected. This information is vital in determining the underpinnings of multistressor responses, which are fundamental in forecasting species responses to changing environments and management responses.

## Graphical abstract

Highlights

- Stress tolerance and resistance were context‐dependent between *T. absoluta* life stages
- Cold hardening showed beneficial acclimation effects on cold tolerance
- Fasting and desiccation pre‐treatments improved survival in adults but not larvae
- Context‐dependent integrated stress resistance may promote *T. absoluta* invasion

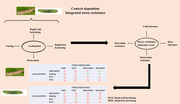

## Introduction

Anthropogenic climate change has been increasingly associated with greater temperature means and variability (Stillman, 2019; Xu *et al*., 2020). However, increased global temperature extremes can also elicit additional abiotic environmental stressors, such as desiccation and food limitation (Nguyen *et al*., 2017) in natural and managed ecosystems. These stressors are most pervasive for terrestrial arthropods, where an ability to invest in compensatory mechanisms is fundamental for fitness under changing environments (Kelley, 2014; Renault *et al*., 2015; Kalra *et al*., 2017). These compensatory responses include basal and plastic thermal tolerance (Kelley, 2014), mutualistic interactions with the biotic environment, metabolic flexibility and others (reviewed in Wan & Yang, 2016; Smit *et al*., 2021), as well as integrated stress resistance (ISR) (see e.g., Renault *et al*., 2015; Gotcha *et al*., 2018; Hector *et al*., 2021; Ricciardi *et al*., 2021). For pest insect invasions in particular, the success of a single or few propagules (i.e., individuals) (Lockwood *et al*., 2005) can potentially lead to colonization by an entire population, necessitating understandings of interactive tolerances to different stressors across life stages. Indeed, these interactive stress tolerances (Renault *et al*., 2015) and other physiobiotic interactions (e.g., Shigesada & Kawasaki, 1997; Mack *et al*., 2000; Worner & Gevrey, 2006; Hill *et al*., 2016; Lu *et al*., 2016) may determine the fate of species establishment in novel environments.

As invasive insect pests are moved from their native regions into novel ranges, they often experience various stressors concurrently (Renault *et al*., 2015), such as temperature extremes and the temporary deprivation of food or water (Mitchell *et al*., 2017; Nguyen *et al*., 2017). Their responses to interactive stressors may manifest in additive (linear effects) synergistic or antagonistic (nonlinear) ways (Todgham & Stillman, 2013; Cheng *et al*., 2015). One of the mechanisms that may elicit nonlinear effects is cross‐tolerance—a physiological adaptation where tolerance to one environmental stressor results in increased tolerance to another divergent stress (Sinclair *et al*., 2013; Cheng *et al*., 2015). Conversely, nonlinear effects may be derived from cross‐susceptibility, where an exposure to one stressor may have no or negative response effect on a divergent stress (Sinclair *et al*., 2013; Nguyen *et al*., 2017). Common mechanisms underlying tolerance to different types of stress include heat shock proteins (Hsps) expression (Lee *et al*., 2004; Gracey *et al*., 2008; Kelley, 2014) or shared regulatory and signalling pathways that activate separate mechanisms of protection against different stresses, that is, “cross‐talk” (Sinclair *et al*., 2013; Renault *et al*., 2015). While such mechanisms are generally exhibited in response to thermal stress, increasing evidence suggest their expression can be in response to other nonthermal stress factors, for example, desiccation and starvation stressors (Eckwert & Köhler, 1997; Köhler *et al*., 2000; Haap & Köhler, 2009; Jiang *et al*., 2012; Haap *et al*., 2016; Augustyniak *et al*., 2017).

Cross‐tolerance is thought to be widespread in insects, particularly with regards to environmental stressors that concomitantly occur. In seasonally and temporally varying environments, insects often survive suboptimal climatic conditions through within and across generational acclimation to different environmental stressors (Chown & Nicolson, 2004; Chown *et al*., 2011; Harrison *et al*., 2012; Sgró *et al*., 2016; Kalra *et al*., 2017). For example, studies have shown positive correlations between resistance to cold and desiccation, as well as between resistance to heat and desiccation stress (Bayley *et al*., 2001; Wu *et al*., 2002, Phelan *et al*., 2003, Bubliy & Loeschcke, 2005; Hayward *et al*., 2007; Singh & Prasad, 2020). In these cases, both stressors result in similar injuries and physiological challenges (see Bayley *et al*., 2001) and potentially promote upregulation of different tolerance mechanisms (Feder & Hofmann, 1999; Kostal *et al*., 2007; Rinehart *et al*., 2007).

Integrated resistance to environmental stress traits, for example, cross‐tolerance, has been observed in numerous invasive insect pests. For example, the lesser mealworm *Alphitobius diaperinus* (Coleoptera: Tenebrionidae), a stored product beetle pest, showed cross‐tolerance between traits of desiccation and cold stress (Renault *et al*., 2015). This ISR is believed to have facilitated its successful establishment in temperate areas such as Europe (Klejdysz & Nawrot, 2010). Another stored product pest, the Khapra beetle, *Trogoderma granarium* is among the most cold‐hardy insect pests, despite originating from hot and dry regions of the Indian subcontinent (Fields, 1992; Wilches *et al*., 2017; Shivananjappa *et al*., 2020). Studies conducted on this pest suggest that the physiological mechanisms that protect diapausing larvae from desiccation may also increase cold tolerance, even though these insects may rarely be exposed to extreme low temperatures in their native environment (Shivananjappa *et al*., 2020). Similarly, desiccation and fasting acclimation improved cold tolerance in invasive spotted stalk borer *Chilo partellus* (Mutamiswa *et al*., 2018). However, ISR may differ with species and thus the interactive effects of one stress on different phenotypes for a single species cannot be generalized across different taxa. Thus, more studies are needed to determine the ISR in insects, and particularly for high‐impact invasive species with a growing global distribution.

The highly invasive tomato leaf miner *Tuta absoluta* Meyrick (Lepidoptera: Gelechiidae) has rapidly spread throughout the globe and successfully established in both temperate and tropical regions (Desneux *et al*., 2010; Biondi *et al*., 2018; Zhang *et al*., 2020). Mechanisms behind its successful invasion are largely speculative, with the exception of a few empirical studies (see e.g., Lietti *et al*., 2005; Roditakis *et al*., 2015; 2018; Wan & Yang, 2016; Machekano *et al*., 2018). This pest possesses several characteristics that promote insect invasion success (see Kelley, 2014; Wan & Yang, 2016), for example, a high propensity to develop pesticide resistance (Lietti *et al*., 2005; Roditakis *et al*., 2015; 2018; Wan & Yang, 2016), phenotypic plasticity (Tarusikirwa *et al*., 2020a), high basal thermal tolerance (Machekano *et al*., 2018), facultative diapause (de Campos *et al*., 2020), host switching (Bawin *et al*., 2016), and high propagule pressure (Guillemaud *et al*., 2015). However, even though *T. absoluta* has successfully established in strikingly heterogeneous environments, no studies have examined the potential for ISR to mediate its invasion dynamics. Cognizant of its economic importance, it thus remains unknown how multiple environmental stressors may influence its physiological fitness in the face of ongoing climate change. Here, we assessed the ISR of *T. absoluta* traits concerning cold‐critical thermal minima (CT_min_), heat‐critical thermal maxima (CT_max_), desiccation, and starvation, following acclimation to each respective stressor. While previous studies have reported on the costs and benefits of acclimation as well as its variation among related species (e.g., Gibert *et al*., 2001; Mitchell *et al*., 2011), we hypothesized that acclimation to one stress may offer protection to a totally divergent stressor (i.e., cross‐tolerance) (see Sinclair *et al*., 2013; Gotcha *et al*., 2018). If found, this phenomenon may explain the global invasiveness of *T. absoluta* in diverse environments, thus informing predictive models and pest management strategies.

## Materials and methods

### Insect culture

Natural populations of *T. absoluta* larvae were collected between April and June 2021 from infested tomato plants at Gurus Farm (S23°7′58.6614″; E27°30′51.6042″), Machaneng village in the Central District of Botswana. In the laboratory, larvae were maintained according to Tarusikirwa *et al*. (2020a), that is, fed on pesticide‐free fresh tomato leaves (“Rodade”) inside insect rearing cages (34 × 34 × 61 cm, Mad Honet, South Africa) under optimum conditions, that is, 28 °C; 65% ± 10% relative humidity (RH) and 12 L : 12 D photocycle in climate chambers (HPP 260; Memmert GmbH + Co. KG, Germany). Acclimation and hardening pretreatments were conducted at slightly different ages within the different life stages, because they lasted different durations (see Table 1 and below), and were randomized across treatments. This thus allowed for stress resistance tests to be conducted on insects of the same age, that is, 3rd‐instar larvae and 6‐day‐old adults (eclosed from field collected larvae). Accordingly, 3rd‐instar larvae and 6‐day‐old adults were tested across all stress tests. Larval instar stage was determined by measuring body length and head capsule, following methods by Rasheed *et al*. (2018) (see Table 2).

**Table 1:** Pretreatment age for *T. absoluta* larvae and adults for the pretreatments (i.e., control, desiccation, fasting, RCH, RHH)

	Age	
Pretreatment	Larvae	Adults
Control	3rd instar	6 days
Desiccation	3rd instar	4 days
Fasting	2nd instar	1 day
RCH	3rd instar	6 days
RHH	3rd instar	6 days

Note: Pretreatment age timing was chosen to allow equilibration of age at the time of stress assays for both larvae and adults to avoid confounding effects of size/age on stress resistance.

RCH, rapid cold hardening; RHH, rapid heat hardening.

**Table 2:** *Tuta absoluta* instar body length and head capsule measurements (Rasheed *et al*., 2018)

Instar stage	Body length (mm)	Head capsule (mm)
2nd	2.66 ± 0.04	0.30 ± 0.03
3rd	4.22 ± 0.06	0.38 ± 0.03
4th	7.59 ± 0.09	0.58 ± 0.04

### High‐temperature hardening

Third‐instar larvae and 6‐day‐old moths were placed in 30 mL plastic vials (*n* = 10) before undergoing hardening experiments. Hardening experiments were performed using established experimental protocols (e.g., Mutamiswa *et al*., 2018; Tarusikirwa *et al*., 2020a). Rapid heat hardening temperature was developmental stage‐dependant and was derived from CT_max_ and defined as 7 °C below CT_max_ (see e.g., Nyamukondiwa *et al*., 2011; Mutamiswa *et al*., 2018) (i.e., 41.7 °C for larvae; 38.5 °C for adults). This hardening temperature is generally adequate to elicit an RHH response in similar insects (Hoffmann *et al*., 2003). The insects were first temperature equilibrated in a climate chamber at 28 °C and 65% ± 10% RH for 30 min before being hardened for 1 h at the high temperature inside a climate chamber set at 41.7 °C (larvae) and 38.5 °C (adults). Following hardening, organisms were given 1 h recovery time (with access to food ad libitum) before measuring stress traits. Control organisms were maintained at optimum conditions (28 °C; 65% RH) before measuring stress traits. A total of 100 organisms per life stage were hardened for both larvae and adults.

### Low‐temperature hardening

Third‐instar larvae and 6‐day‐old moths were placed in 30 mL plastic vials (*n* = 10). Hardening experiments were performed using established experimental protocols (following e.g., Mutamiswa *et al*., 2018; Tarusikirwa *et al*., 2020a). Cold hardening temperature was derived from CT_min_ and was defined as 6 °C below CT_min_ (Nyamukondiwa *et al*., 2011; Mutamiswa *et al*., 2018) (i.e., −4 °C for larvae; −6 °C for adults). These hardening temperatures are sufficient to elicit RCH responses in similar organisms under laboratory experimental conditions (Lee & Denlinger, 2010). Larval and adult *T. absoluta* specimens were first placed in a climate chamber set at 28 °C and 65% ± 10% RH for 30 min to allow for equilibration. Thereafter, the insects were placed in a zip lock bag and submerged in a programmed water bath (Systronix Scientific, Industria, South Africa) with 1 : 1 water : propylene glycol and hardened for 1 h. Following treatment, insects were allowed to recover under benign conditions (28 °C; 65% ± 10% RH) in a climate chamber for 1 h to allow the *de novo* Hsps synthesis and with access to food ad libitum (Hoffman *et al*., 2003) before measuring stress traits. Control insects were kept at benign conditions during the same treatment before measuring stress traits. A total of 100 organisms per life stage were hardened for both larvae and adults.

### Desiccation acclimation

Third‐instar larvae and 4‐day‐old moths were individually placed in 30 mL perforated plastic vials (*n* = 10). Acclimations were done with different age groups to facilitate testing stress resistance traits for similar developmental stages at both larval and adult stages, given different pretreatment lengths (see Table 1). The vials were then placed in a modified humidity inside a desiccator. Humidity was modified to 1% ± 1% RH by placing 150 g of silica gel inside the desiccator (desired humidity was reached after ± 15 min). The desiccator was then placed in a climate chamber set at 28 °C for a period of 24 h. This duration was sufficient to elicit 40% body water loss in test insects (e.g., see Tarusikirwa *et al*., 2020b). Thermocron iButtons (Dallas Semiconductors, Model DS1920) were used to monitor the temperature and RH inside the desiccator at 30‐min intervals. After 24 h of desiccation stress, the organisms were removed from the desiccator and given a recovery period of 24 h with access to food and water ad libitum at 28 °C, 65% RH before stress tests. The control organisms were maintained under benign conditions before conducting stress tests. A total of 100 organisms per life stage were subjected to desiccation acclimation for both larvae and adults following methods by Gotcha *et al*. (2018).

### Starvation acclimation

Second‐instar and 2‐day‐old moths were placed in 30 mL perforated plastic vials (*n* = 10). As mentioned above (“desiccation acclimation” section), acclimations were done with different age groups to facilitate testing stress resistance traits for similar developmental stages at both larval and adult stages, given different pretreatment lengths (see Table 1). A damp cotton wad was placed inside the vial to prevent mortality associated with desiccation (Parkash *et al*., 2014). The organisms were then placed in a climate chamber under optimum conditions (i.e., 28 °C; 65% RH) for 48 h (larvae) and 72 h (adults), with no access to food, that is, “fasting acclimation.” Preliminary acclimations for the two developmental stages showed that this is the optimal time required to elicit positive acclimation responses and avoid mortality due to prolonged pretreatment stress. Thus, the starvation acclimation timing was different between the two developmental stages. After the respective acclimation durations of food deprivation, the organisms were given a 24 h recovery period (provided with water and food ad libitum, at optimum conditions). Control organisms were sorted in the same way as treatment organisms but were placed in 30 mL vials with ad libitum access to food (tomato leaves for larvae and 10% sugar solution for adults) under optimum conditions for subsequent stress tests. A total of 100 organisms per life stage were subjected to fasting pretreatment for both larvae and adults.

### Stress tolerance and resistance tests

For all hardening and acclimation experiments, insects were exposed to sublethal stressful conditions that would elicit acclimatory responses without mortality, that is, without allowing for selection (see Mitchell *et al*., 2011). After subjecting the organisms to different environmental stress acclimation conditions (i.e., control, RHH, RCH, desiccation, fasting), they were exposed to the same (but more severe) and other divergent stress tolerance/resistance tests to measure tolerance and resistance to high‐ and low‐temperature stress tolerance, desiccation and starvation, respectively, that is, CT_max_, CT_min_, desiccation resistance, and starvation resistance. Both CT_max_ and CT_min_ are ecologically relevant traits of thermal tolerance (Nyamukondiwa & Terblanche, 2009; Andersen *et al*., 2015; Gotcha *et al*., 2018) and have been used as reasonable proxies of high‐ and low‐temperature tolerance, respectively (but see discussions in Clusella‐Trullas *et al*., 2021). The staggering of these stress resistance traits facilitated uniformity with those of critical thermal limits, that could only be done using *n* = 10 per time because of limitations of the organ pipes. Furthermore, interactions across these acclimations for the four stress traits assayed here were also measured (see Table 3).

**Table 3:** Binomial generalized linear mixed model results considering (a) desiccation and (b) starvation effects on survivability

Trait	Effect	*χ* ^2^	df	*P* value
(a) Desiccation	**Stage**	**7.756**	**1**	**0.005**
	Treatment	5.517	4	0.238
	**Duration**	**175.019**	**1**	**<0.001**
	**Stage × Treatment**	**10.057**	**4**	**0.040**
	**Stage × Duration**	**5.552**	**1**	**0.019**
	Treatment × duration	1.839	4	0.765
	**Stage × Treatment × Duration**	**13.041**	**4**	**0.011**
(b) Starvation	**Stage**	**9.865**	**1**	**0.002**
	**Treatment**	**12.652**	**4**	**0.013**
	**Duration**	**180.774**	**1**	**<0.001**
	**Stage × Treatment**	**79.100**	**4**	**<0.001**
	**Stage × Duration**	**7.761**	**1**	**0.005**
	**Treatment × Duration**	**18.479**	**4**	**<0.001**
	Stage × Treatment × Duration	7.777	4	0.100

Note: Terms are computed stepwise following backward deletion of nonsignificant effects, where applicable. Significant terms are emboldened.

#### Critical thermal maxima

CT_max_ was measured on *T. absoluta* larvae and adults using standardized protocols as outlined by Nyamukondiwa and Terblanche (2009). Ten replicate organisms were individually placed in a series of numbered “organ pipes” connected to an insulated double‐jacketed chamber which was linked to a programmable water bath before ramping up the temperature from a setpoint temperature of 28 °C (10 min) at a rate of 0.25 °C/min until their CT_max_ were recorded. This was repeated twice for each trait measured to yield a total sample of 20 individuals (*n* = 20), where each individual served as a replicate. The organ pipes that measure critical thermal limits (CT_min_ and CT_max_) only take 10 organisms per cycle. Thus, the two repeated hardening cycles of *n* = 10 made it possible to measure critical thermal limits twice, immediately after each acclimation, before hardening effects were reversed (see discussions in Weldon *et al*., 2011). Critical thermal maximum was defined as the upper temperature at which each individual insect lost coordinated muscle function and ability to respond to mild stimuli using a thermally inert object.

#### Critical thermal minima

CT_min_ was measured using the same protocol as that of CT_max_ (Nyamukondiwa & Terblanche, 2009) except that temperature was ramped down from a setpoint temperature of 28 °C (10 min) at a rate of 0.25 °C/min until the CT_min_ endpoint. Critical thermal minima were defined as the lowest temperature at which each individual insect lost coordinated muscle function and ability to respond to mild stimuli using a thermally inert object. This was replicated twice for each trait measured (*n* = 20). The two repeated hardening cycles of *n* = 10 also made it possible to measure critical thermal limits twice, immediately after each acclimation, before hardening effects were reversed (Weldon *et al*., 2011).

#### Desiccation resistance

Following the recovery period from the various acclimation pretreatments, organisms were placed in desiccators with modified humidity, 1% ± 1% RH (150 g silica gel). Thirty replicate organisms were used for each pretreatment (*n* = 30). Thereafter, the desiccators with test organisms were transferred and maintained at optimum temperature (28 °C) for the entire duration of the experiment in a climate chamber. Control organisms were maintained in a climate chamber under benign conditions, that is, 28 °C, 65% ± 10% RH. Organisms were checked for mortality after 24, 48, 72, and 96 h intervals (i.e., individuals were repeatedly measured). Desiccation resistance was thus examined with respect to mortality rates following these different desiccation exposure durations. This was replicated thrice for each trait measured (*n* = 30).

#### Starvation resistance

Thirty replicate insects for each developmental stage exposed to pretreatments were used for starvation resistance tests. Both treated and control organisms were placed into 30 mL perforated plastic vials. The vials were then placed inside a climate chamber under optimum conditions and monitored for 4 days (*n* = 30 pretreatment). To avoid mortality associated with desiccation while “starving,” moist cotton wad was placed inside each vial (see Bubliy *et al*., 2012; Parkash *et al*., 2014). Mortality was assessed after every 24 h for 4 days—a period in which most larvae were either dead or had pupated (survived) (Gotcha *et al*., 2018).

### Data analyses

Differences in CT_max_ and CT_min_ for the five treatments were examined using separate Kruskal–Wallis tests, each for larval and adult stages, as data violated assumptions of parametric tests. Dunn tests were used for post hoc pairwise comparisons in cases where treatment was found to be significant (Ogle *et al*., 2020).

Factorial binomial generalized linear mixed models with logit links were used to examine the effects of life stage, treatment and duration, separately according to the desiccation and starvation survivability experiments (Brooks *et al*., 2017). Each individual replicate was included as a random effect to account for repeated measures through time. In each model, stepwise reductions were used to remove nonsignificant terms until the final model included only significant predictors, with Type III analysis of deviance used to compute coefficients in the final model (Fox & Weisberg, 2019). Estimated marginal means were used to compute post hoc tests for significant terms in generalized linear models (Length, 2020). All analyses were computed in R v4.0.2 (R Core Team, 2020).

## Results

### Thermal tolerance

**Fig. 1:**
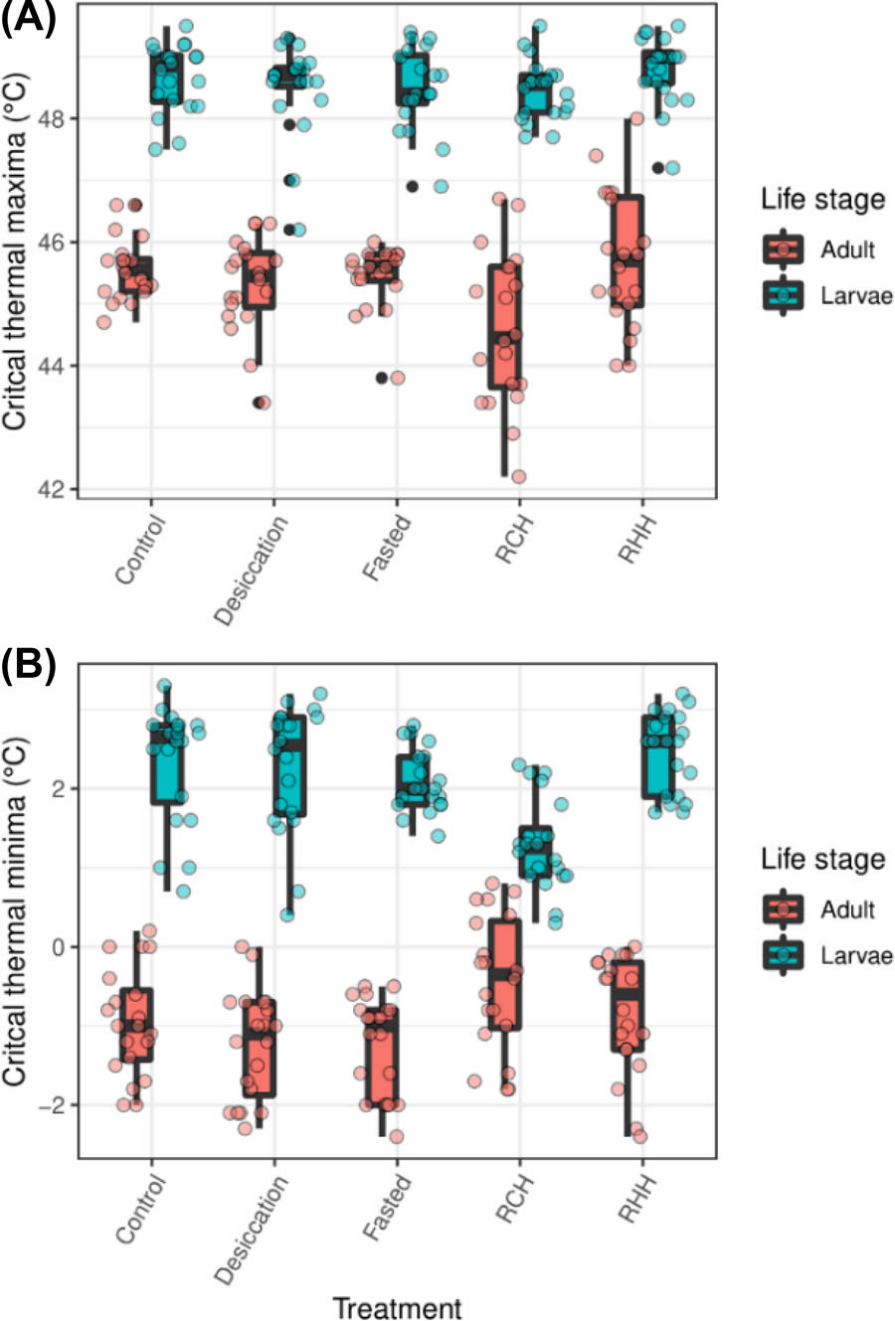
Critical thermal maxima (A) and minima (B) according to treatment (desiccation and fasting acclimation, rapid cold hardening [RCH] and rapid heat hardening [RHH] and life history stage). In the boxplots, the horizontal bars display the median, the box gives the interquartile ranges, and the whiskers show the largest and smallest values up to 1.5× interquartile range.

There was no significant improvement in heat tolerance in both life stages following the various acclimation pretreatments compared to controls (all *P* > 0.05), that is, RHH, RCH, desiccation, and starvation (Fig. 1A). In adults, however, heat tolerance (CT_max_) differed significantly according to treatment (*χ* ^2^ = 10.286, df = 4, *P* = 0.036; Fig. 1A), such that RCH significantly reduced CT_max_ compared to RHH (*P* = 0.043). No other treatments were significantly different pairwise (all *P* > 0.05) (Fig. 1A). Conversely, treatment did not have a significant effect on CT_max_ at larval stages (*χ* ^2^ = 6.607, df = 4, *P* = 0.158), and thus all treatment pairs were statistically similar (all *P* > 0.05), but these were significantly higher than adults (mean range adults: 44.590–45.705 °C; mean range larvae: 48.490–48.805 °C; *χ* ^2^ = 147.610, df = 1, *P* < 0.001; Fig. 1A).

In adults, treatments did not improve cold tolerance (CT_min_) as no treatment groups significantly differed from the control (all *P* > 0.05) (Fig. 1B). Critical thermal minima for adults, however, again differed significantly among treatments (*χ* ^2^ = 12.527, df = 4, *P* = 0.014; Fig. 1B). The RCH‐treated adults exhibited significantly reduced cold tolerance (i.e., higher CT_min_ temperature) compared to desiccated (*P* = 0.030) and fasted (*P* = 0.028) ones. Larvae also exhibited significant differences in CT_min_ (*χ* ^2^ = 29.629, df = 4, *P* < 0.001). The RCH‐treated larvae exhibited significantly greatest cold tolerance (i.e., lowest CT_min_ temperature) compared to all other treatments and controls (all *P* < 0.05) (Fig. 1B). All other treatment pairs were statistically similar (all *P* > 0.05). Adults were significantly more cold tolerant than larvae overall (mean range adults: −0.450 to −1.255 °C; mean range larvae: 2.465–1.290 °C; *χ* ^2^ = 148.28, df = 1, *P* < 0.001; Fig. 1B).

### Desiccation and starvation resistance

**Fig. 2:**
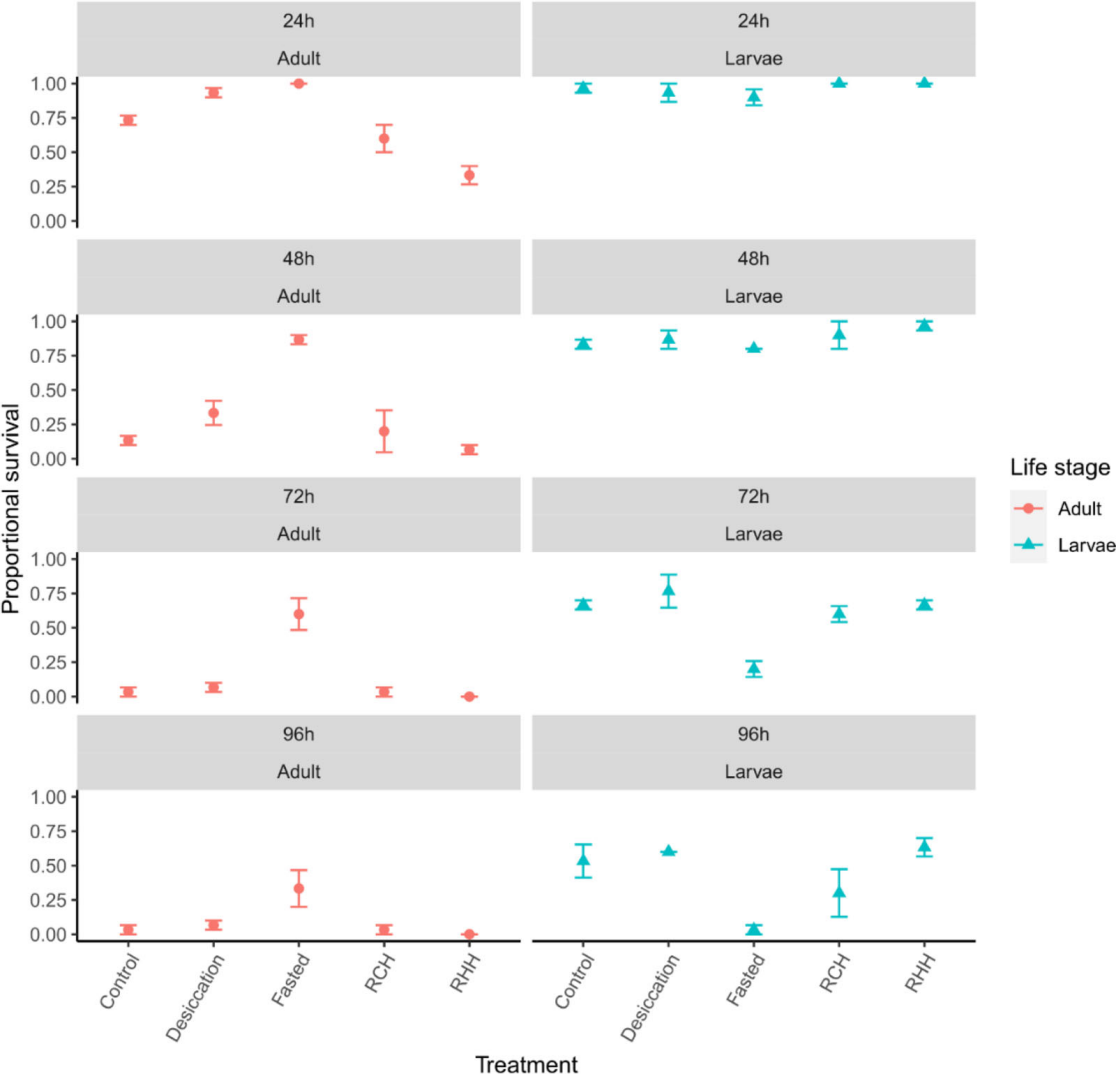
Mean (± standard error) survival to desiccation between adult and larval life stages, following exposure to various prior stressor treatments (desiccation and fasting acclimation, rapid cold hardening [RCH] and rapid heat hardening [RHH]) and following different durations.

For desiccation resistance, the effects of pretreatment significantly interacted with life stage and exposure duration in driving mortality (Table 3 and Fig. 2). In adults, the fasted group exhibited significantly greater desiccation resistance than controls (*P* < 0.001) (Fig. 2). Desiccated, RHH‐, and RCH‐treated adults were more similar to controls (both *P* > 0.05). Fasted adults thus showed significant cross‐tolerance, additionally exhibiting greater survival than desiccated, RCH‐, and RHH‐treated adults (all *P* < 0.001). In larvae, fasted groups, conversely to adults, exhibited significantly lowest survival (all *P* < 0.01). All other groups were statistically similar to the controls (*P* > 0.05). Survival trends over time did not differ according to treatment in adults (all *P* > 0.05), whereas fasted larval survival fell most sharply with increasing exposure duration, and significantly more so than desiccated individuals (*P* = 0.018) and borderline significantly with controls (*P* = 0.051).

**Fig. 3:**
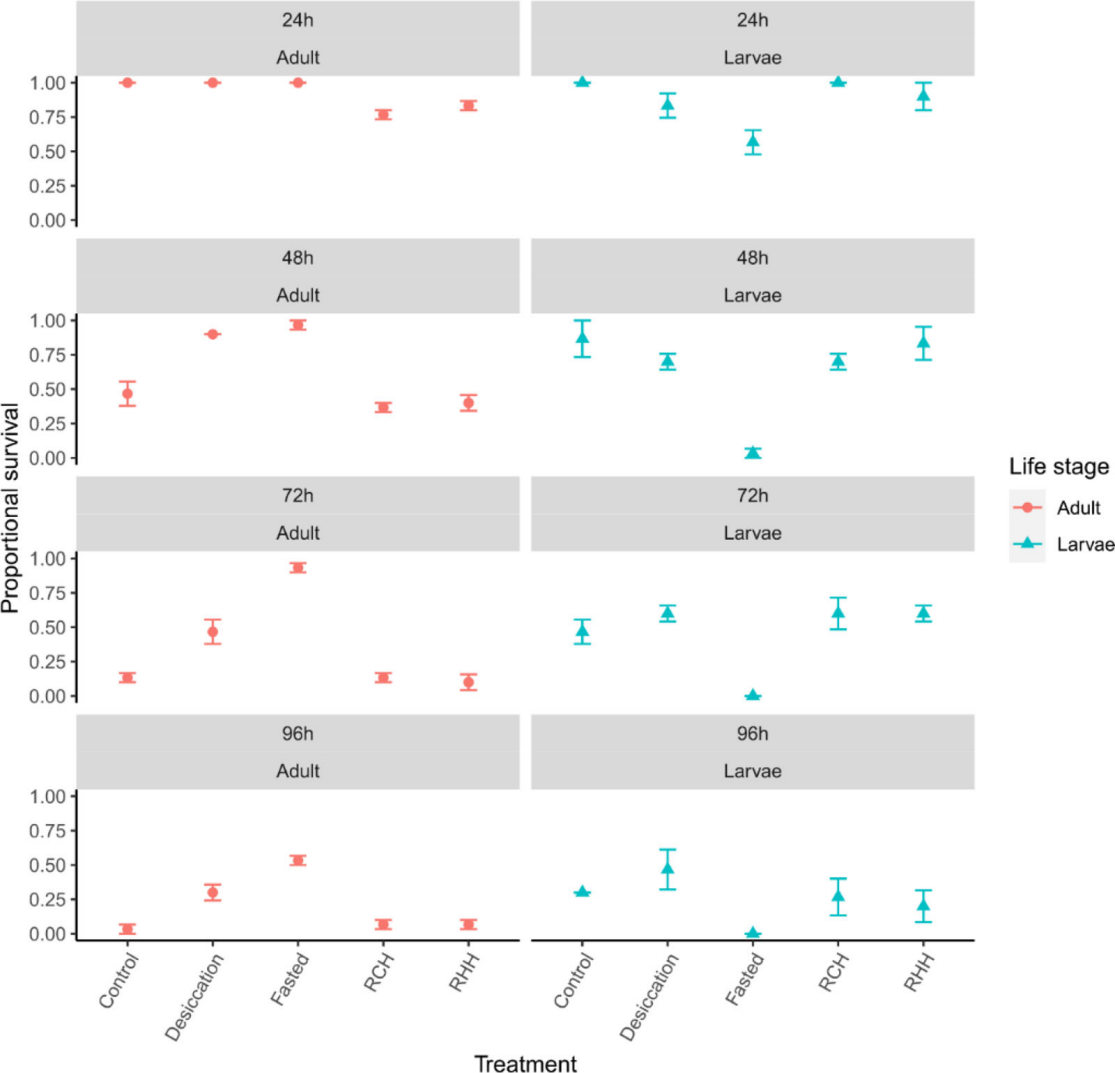
Mean (± standard error) survival to starvation between adult and larval life stages, following exposure to various prior stressor treatments (desiccation and fasting acclimation, rapid cold hardening [RCH] and rapid heat hardening [RHH]) and following different durations.

Similar to desiccation, starvation‐induced resistance was significantly affected by treatment in interaction with life stage, but there was no significant three‐way interaction with exposure duration (Table 3 and Fig. 3). For adults, fasted and desiccated groups exhibited significantly greater survival than controls (both *P* < 0.001), while all other groups were statistically similar to controls (all *P* > 0.05) (Fig. 3). Fasted adults also exhibited significantly higher survival than desiccated, RCH‐, and RHH‐treated ones (all *P* < 0.001), while desiccated individuals also survived significantly better than RCH‐treated adults (*P* < 0.001) and RHH‐treated adults (*P* < 0.001); RCH and RHH were statistically similar (*P* > 0.05). For larvae, conversely to adults, fasted individuals exhibited significantly lower survival than controls (*P* < 0.001), with all other treatment groups statistically similar to controls (all *P* > 0.05). Fasted groups also exhibited significantly lower survival following starvation compared to all other groups (all *P* < 0.001), with those other groups statistically similar (all *P* > 0.05). Accordingly, starvation conferred an adaptive advantage to adults after starvation exposure, but a disadvantage to larvae. Duration significantly affected survivability in interaction two‐way with both treatment and life stage (Table 3 and Fig. 3).

## Discussion

Cross‐tolerance likely shows shared coevolved response mechanisms to totally contrasting environmental stressors. This ISR likely saves energetic costs associated with the mounting of divergent stress responses for cooccurring environmental pressures and is likely a key factor that promotes fitness and invasiveness in pest insects (Renault *et al*., 2015). Our results show novel context‐dependence between life stages, with certain prior stressors imparting an advantage to adults but a disadvantage to larvae. Principally, we show that prior cold thermal stresses influence CT_max_ and CT_min_ in adults and larvae, but not for desiccation and starvation resistance. This resonates well with the rejection of the beneficial acclimation hypothesis (BAH) for short‐term stress responses (Ramniwas *et al*., 2020). Second, we show that fasting improves desiccation tolerance in adults (cross‐tolerance), while showing a negative effect (cross‐susceptibility) in larvae. Similarly, desiccation acclimation also improved adult starvation tolerance, indicating significant cross‐tolerance effects. This shows that interaction between divergent stressors may be dependent on the developmental stage context. Third, we show that acclimation to one stress may come at a cost of another divergent environmental stress (cross‐susceptibility). For example, RCH came at a cost of heat tolerance (CT_max_) in adults and that RCH was maladaptive for CT_min_.

This study provides insights on developmental stage related responses to multiple environmental stressors and the interaction thereof in *T. absoluta*. Acclimation to one particular environmental stressor may provide survival advantages upon exposure to the same but more stressful environment, termed BAH (Wilson & Franklin, 2002). Our results generally show lack of support for BAH as well as differential developmental stage resistance and thermal tolerance following exposure to environmental stresses. This result is in consonance with recent literature debating the BAH and the rejection thereof for short‐term plastic responses (Raminwas *et al*., 2020). Indeed, RHH had no significant effects on CT_max_, a result that is in keeping with *Zygogramma bicolorata* (Chidawanyika *et al*., 2017) and *Drosophila nepalensis* (Raminwas *et al*., 2020). When different stressors activate distinct molecular pathways, exposure to one may have no effect on response to the other contrasting stress or may come at a cost due to the energetic demands of responding to multiple stressors simultaneously (Sinclair *et al*., 2013; Todgham & Stillman, 2013; Nguyen *et al*., 2017). While RHH, desiccation, and fasting acclimation did not have significant effects on heat tolerance in both larvae and adults, RCH had a significant cost to adult CT_max_ indicating cross‐susceptibility effect. This result is in agreement with reports on related lepidopteran species: *C. partellus*, *Busseola fusca*, and *Sesamia calamistis* (Mutamiswa *et al*., 2018). Similarly, dehydration acclimation and RHH had no effects on *Cimex lectularius* heat tolerance (Benoit *et al*., 2009; Everatt *et al*., 2014). However, our results are in contrast with the notion that episodes of heat stress often coincide with water restriction, and thus responses to either heat or desiccation may promote resistance to both stresses (Renault *et al*., 2015). A comparison of both life stages showed larvae exhibited higher CT_max_ than adults following all treatments, indicating that larvae were more heat tolerant than adults. Exact mechanisms for this difference are unknown and warrant further investigation. However, larvae have higher body water‐ and lipid‐content than adults (see Tarusikirwa *et al*., 2021). This may likely point to the role of body water‐ and lipid‐content in heat tolerance (Huey & Kearney, 2020), and might favor the larval role for invasion success in heat‐stressed environments.

None of the pretreatments, that is, RHH, starvation, and desiccation, elicited a cross‐tolerance response to low‐temperature (CT_min_) tolerance in adults compared to controls. Rapid cold hardening had, however, significant beneficial effects on larval CT_min_ while it was significantly maladaptive for adult cold tolerance compared to prior starvation and desiccation. This result suggests that acclimation to low temperature is developmental‐dependent, in keeping with Mutamiswa *et al*. (2019). The cross‐susceptibility between RCH and cold tolerance (CT_min_) in adults suggests that RCH may come at a cost of low‐temperature tolerance in adult *T. absoluta*, likely owing to the additive thermal injury associated with both acclimation and stress exposure (Jørgensen *et al*., 2021). This result is in agreement with Tarusikirwa *et al*. (2020a) and Keosentse *et al*. (2021), who reported differences in RCH between larval and adult *T. absoluta* and *Spodoptera frugiperda*, respectively. While not found here, there is a close association between desiccation and cold such that dehydration stress often confers improved cold tolerance (Yi *et al*., 2017); for example, exposure to mild desiccation was reported to increase cold tolerance in the springtail, *F. candida* (Bayley *et al*., 2001).

Fasting reduced cold tolerance in *Tribolium castaneum* (Coleoptera: Tenebrionidae) (Scharf *et al*., 2015). Similarly, Hoffmann *et al*. (2005) showed that flies selected for starvation resistance had reduced resistance to cold, whereas those selected for increased cold resistance show decreased starvation resistance (Singh & Prasad, 2020). Here, a comparison of *T. absoluta* larvae and adults showed adults had higher cold tolerance (more negative CT_min_ temperature) than larvae across all treatments. This suggests that physiological responses to environmental stressors in nature are life stage‐ and trait‐dependent (Mutamiswa *et al*., 2019) and shows that different developmental stages may select for synergistic resistance to different stressors under natural conditions. Results also showed no significant effects of RHH for CT_min_. Mechanisms required to adapt to a specific type of stress might conflict with mechanisms required to adapt with other kinds of stress, leading to trade‐offs across stress resistance traits (Kellett *et al*., 2005; Overgaard *et al*., 2006; Macmillan *et al*., 2009; Scharf *et al*., 2015). This may also mean that mechanisms for heat and cold resistance are decoupled, and that stressful and injurious effects between RHH and cold tolerance may be additive (Jørgensen *et al*., 2021).

One striking result of the current study is that fasted adults had significantly higher desiccation tolerance, while the same acclimation effect was maladaptive in larvae. Therefore, stresses associated with translocation during early invasion stages may mean that invader “propagules” arriving in a novel environment may have already been exposed to stressors, which promote their survival in new environments. Similarly, transportation of aquatic invaders in ships’ ballast water can stress populations and potentially select for individuals that are more invasive (Briski *et al*., 2018). In insects, organisms deprived of food during anthropogenically associated translocation, may thus have improved desiccation tolerance—a positive cross‐tolerance effect that may improve survival in arid environments (Renault *et al*., 2015). Studies by Raubenheimer and Gäde (1993) showed that there is a possible link between the reduction in lipid levels and an increase in water uptake. Generally, there is a decrease in lipid levels following starvation; this can lead to an increase in water stores available for the insect to utilize in withstanding desiccation stress (Mitchell *et al*., 2017). However, other acclimation pretreatments had no effect on desiccation tolerance between both developmental stages. This is again in agreement with Bubliy *et al*. (2012), who reported cross‐susceptibility between traits of cold acclimation on desiccation resistance of adult female *D. melanogaster*. However, the result is also contrary to Gotcha *et al*. (2018) who reported improved desiccation resistance following cold acclimation adult *C. rosa* of mixed sex, suggesting context complexity in teasing cross‐tolerance/ISR effects.

A comparison between larvae and adults showed larvae had significantly higher desiccation resistance across all durations and following acclimations. This implies that larvae are more desiccation resistant than adults, a result resonating well with Tarusikirwa *et al*. (2021). Desiccation acclimation did not significantly improve desiccation tolerance, and as such, we reject the BAH for desiccation tolerance, with caveats, at least for the current methodological context. Nevertheless, this study adds to the current debate on the BAH and the rejection thereof for short‐term stress responses (Ramniwas *et al*., 2020).

Desiccation and fasting acclimation significantly improved starvation resistance in adults, but not so in larvae. This significant ISR between desiccation and starvation tolerance in adults suggest that continuous bouts of low food resources and RH in nature may give a survival advantage in adults that may optimize fitness and invasiveness under anthropogenically changing environments. Given that adults are responsible for propagation and dispersal, this survival advantage may lead to population perpetuation when they encounter increasingly low food resources and desiccation stress under arid tropical environments. Geographic dispersal of *T. absoluta* has been reported to be primarily driven by short and long‐distance movements (Xian, 2017). While international agricultural trade (e.g., imports and exports of infested fruits, propagule material and packaging materials) and natural factors (wind and water) are the key long‐ and short‐distance dispersal mechanisms (Xian, 2017), there is a high likelihood of mobile life stages (larvae and adults) experiencing multiple environmental stressors in transit. Thus, ISR reported here may be adaptive and likely improves *T. absoluta* competitiveness in invaded environments. While this study targeted a single invasive species, future studies may need to focus on cross‐species comparison particularly invaders versus trophically analogous noninvaders to fully elucidate key attributes aiding invasion in insects.

In conclusion, the results first show that acclimation to one particular stressor may confer improved (cross‐tolerance) or impairment/no effect (cross‐susceptibility) to other contrasting stressors, but these effects differ between life stages. For example, fasting improved desiccation tolerance in *T. absoluta* adults (cross‐tolerance), while showing a negative effect (cross‐susceptibility in larvae). Similarly, desiccation (as well as fasting) acclimation improved starvation tolerance in adult *T. absoluta*. This cross‐tolerance may strengthen pest fitness in novel arid and environmentally heterogeneous invaded environments, potentially facilitating invasive pest establishment (Renault *et al*., 2015). Second, we show that acclimation to one particular stress may come at a cost of another divergent environmental stress (cross‐susceptibility), suggesting some stress responses may be additive. Considering that different stressors may affect *T. absoluta* interactively in various possible ways, the results are important in modeling its risk status and developing sustainable management options under changing environments. Incorporating these integrated stress responses into predictive distributional models may help refine invasive pest species potential biogeographical niches and may help safeguard the environment from the damaging effects of alien invasive species.

## Disclosures

All authors have seen and agree with the contents of the manuscript and there is no conflict of interest, including specific financial interest and relationships and affiliations relevant to the subject of manuscript.
